# Assessment of Physiological Rat Kidney Ageing—Implications for the Evaluation of Allograft Quality Prior to Renal Transplantation

**DOI:** 10.3390/metabo12020162

**Published:** 2022-02-08

**Authors:** Andreas Baumgartner, Simone Reichelt-Wurm, Wolfram Gronwald, Claudia Samol, Josef A. Schröder, Claudia Fellner, Kathrin Holler, Andreas Steege, Franz Josef Putz, Peter J. Oefner, Bernhard Banas, Miriam C. Banas

**Affiliations:** 1 Department of Nephrology, University Hospital Regensburg, 93053 Regensburg, Germany; andreas.c.fuchs@gmail.com (A.B.); kathrin.holler@ukr.de (K.H.); andreas_steege@yahoo.de (A.S.); franz-josef.putz@ukr.de (F.J.P.); bernhard.banas@ukr.de (B.B.); 2 Department of Orthopedics and Trauma Surgery, Medical Center-Albert-Ludwigs-University Freiburg, 79106 Freiburg, Germany; 3 Institute of Functional Genomics, University of Regensburg, 93053 Regensburg, Germany; claudia1.bogner@ukr.de (C.S.); peter.oefner@ukr.de (P.J.O.); 4 Institute of Pathology, University of Regensburg, 93053 Regensburg, Germany; josef.schroeder@ukr.de; 5 Department of Radiology, University Hospital Regensburg, 93053 Regensburg, Germany; claudia.fellner@ukr.de

**Keywords:** kidney, ageing, transplantation, inflammation, urinary metabolites

## Abstract

Due to organ shortage and rising life expectancy the age of organ donors and recipients is increasing. Reliable biomarkers of organ quality that predict successful long-term transplantation outcomes are poorly defined. The aim of this study was the identification of age-related markers of kidney function that might accurately reflect donor organ quality. Histomorphometric, biochemical and molecular parameters were measured in young (3-month-old) and old (24-month-old) male Sprague Dawley rats. In addition to conventional methods, we used urine metabolomics by NMR spectroscopy and gene expression analysis by quantitative RT-PCR to identify markers of ageing relevant to allograft survival. Beside known markers of kidney ageing like albuminuria, changes in the concentration of urine metabolites such as trimethylamine-N-oxide, trigonelline, 2-oxoglutarate, citrate, hippurate, glutamine, acetoacetate, valine and 1-methyl-histidine were identified in association with ageing. In addition, expression of several genes of the toll-like receptor (TLR) pathway, known for their implication in inflammaging, were upregulated in the kidneys of old rats. This study led to the identification of age-related markers of biological allograft age potentially relevant for allograft survival in the future. Among those, urine metabolites and markers of immunity and inflammation, which are highly relevant to immunosuppression in transplant recipients, are promising and deserve further investigation in humans.

## 1. Introduction

Donor organ shortage is a major issue in kidney transplantation. With a population steadily ageing, the number of elderly patients on waiting lists for transplantation, but also the age of living organ donors, are rising. To face this issue, allocation programs, such as the Eurotransplant Senior Program [1], have been developed, notably allocating older donor organs to older recipients.

Donor organ quality is essential for a successful long-term outcome in kidney transplantation. Accurately predicting organ quality remains a major challenge [2,3]. Previously many research projects focused on pathological loss of kidney function, e.g., on renal fibrosis secondary to renal damage. However physiological kidney ageing needs to be addressed as the calendric kidney age can differ markedly from its “biologic” age.

Many studies have shown that the age of the donor, whether deceased or alive, along with the age of the recipient, are among the main factors affecting graft and patient survival [4,5,6]. But the chronological age does not always correspond to the biological age of the transplanted organs, which can also be affected by different factors e.g., diabetes, smoking or genetics. A null biopsy from the donated organ before explantation would be ideal, but this is often not feasible due to logistic and time reasons.

Therefore, identifying reliable biomarkers of kidney function in old organ donors may help assist the selection of donors but also the management of recipients after transplantation. Several parameters have been investigated to better assess kidney function, notably in relation with ageing [2,3,7,8,9,10]. Established methods of kidney function assessment are based mainly on histological examination of renal damage and the measurement of conventional markers in blood and urine (e.g., creatinine, albumin, urea) [2,6]. Furthermore, methods based on transcriptomics or proteomics aim at identifying molecular markers associated with biological age as indicators of organ quality [3]. For instance, expression of genes associated with inflammation and immune activation has been correlated with kidney function and with ageing (so-called inflammaging) [8,11,12,13,14,15,16]. However, these studies are still at their infancy and no molecular markers have been validated and implemented in routine clinical practice yet. 

The present study aimed to better characterize age-related markers of kidney function. We used a model of healthy Sprague Dawley rats and applied a panel of both conventional and innovative methods to compare structural, biochemical, and molecular characteristics of young (3-month-old) and old (24-month-old) rat kidneys. We confirmed previous studies showing proteinuria and histological lesions in old animals. We also identified age-related changes in gene expression of potential inflammaging factors using quantitative RT-PCR, and in the concentration of specific urine metabolites using NMR spectroscopy. 

## 2. Results

To identify age-related markers, a series of biochemical, histological, and molecular parameters were compared between 3- and 24-month-old Sprague Dawley rats. We focused on male rats, to avoid sex-related confounding factors. 

### 2.1. Vital and Serum Parameters

Vital parameters (blood pressure and heart rate) were not significantly different between 3- and 24-month-old rats. With the exception of blood urea nitrogen (BUN), uric acid, and glucose, the serum levels of which were significantly lower in older rats, serum levels of conventional markers of kidney function and ageing (creatinine, albumin, total protein, CRP, ASAT, ALAT, cholesterol, triglyceride) did not differ significantly between 3- and 24-month-old rats (Table 1).

### 2.2. Urine Parameters

**Figure 1:**
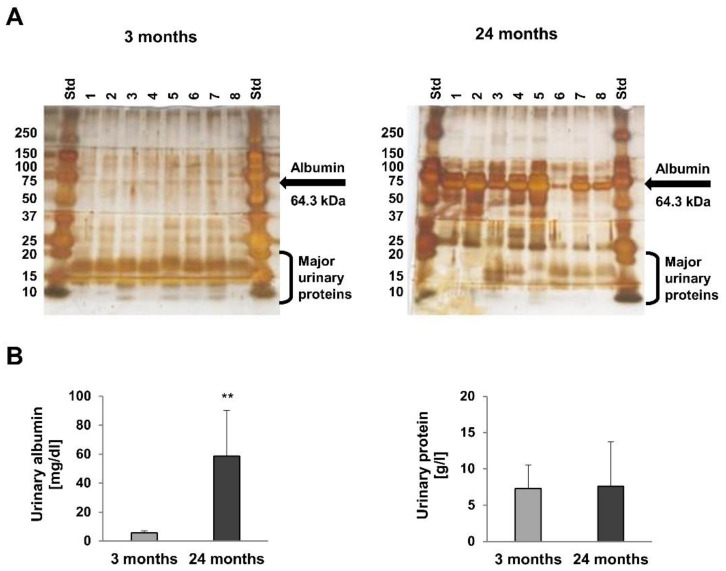
Urine analysis of 3- and 24-month-old rats. High levels of urinary albumin were detected in all eight 24-month-old rats by SDS-PAGE and silver staining (**A**), as well as by ELISA (**B**; *p* = 0.0002). The total protein level in urine was not significantly different at 3 and 24 months of age (**B**; *p* = 0.959). This might be explained by the presence of high levels of major urinary proteins (MUPs) in young male rats, as opposed to older rats (**A**), hence masking the detection of albuminuria in old rats in the BAC assay. ** *p* < 0.01 versus 3 months old rats.

Proteinuria is one of the main manifestations of kidney dysfunction [2,7,17] and was thus evaluated in young and old rats. High levels of urinary albumin were detected in all eight 24-month-old rats by SDS-PAGE and silver staining (Figure 1A), as well as by ELISA (Figure 1B; *p* = 0.0002). the total urinary protein levels did not differ significantly at 3 and 24 months of age (Figure 1B; *p* = 0.959). This might be explained by the presence of high levels of major urinary proteins (MUPs) in young male rats [18], as opposed to older rats (Figure 1A), hence masking the detection of albuminuria in old rats in the BAC assay. Creatinine (68.0 mg/dL vs. 63.2 mg/dL, *p* = 0.721) and urea (36.4 g/L vs. 35.2 g/L, *p* = 0.958) levels in urine were comparable in 3- and 24-month-old rats.

### 2.3. Urine Metabolomics

**Figure 2:**
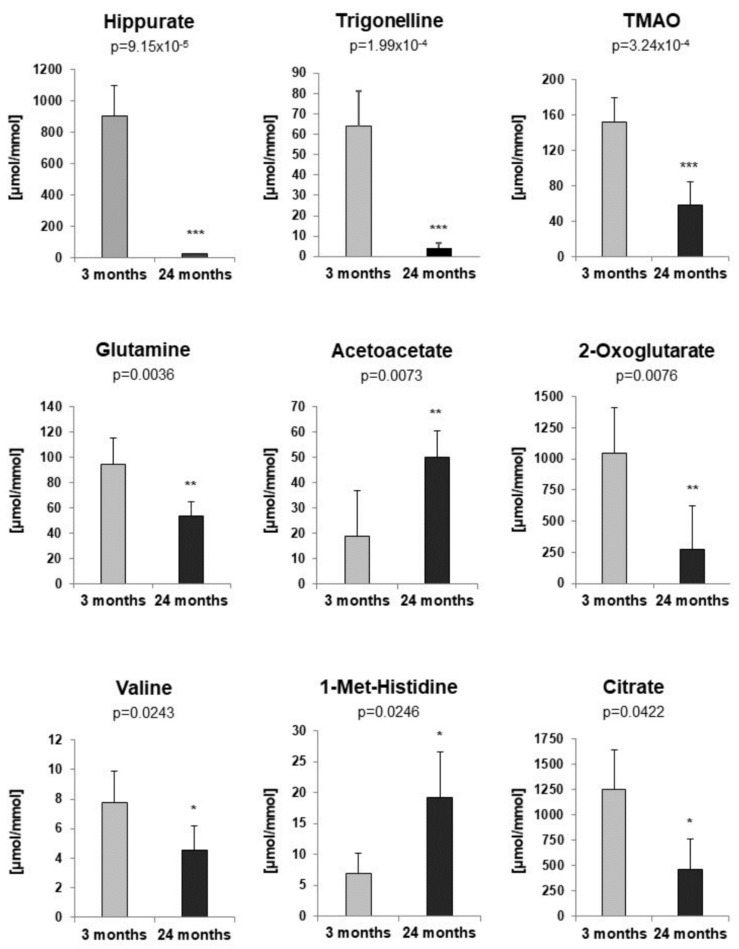
Targeted profiling of urine metabolites of 3- and 24-month-old rats by NMR spectroscopy. All concentrations were normalized to creatinine [µmol/mmol]. *p*-values were determined by a two-sided Welch *t*-test. To account for multiple testing the false discovery rate was controlled at the 5% level. For all figures *p*-values ≤ 0.05, ≤0.01, and ≤0.001 are indicated by one, two or three asterisks, respectively.

**Figure 3:**
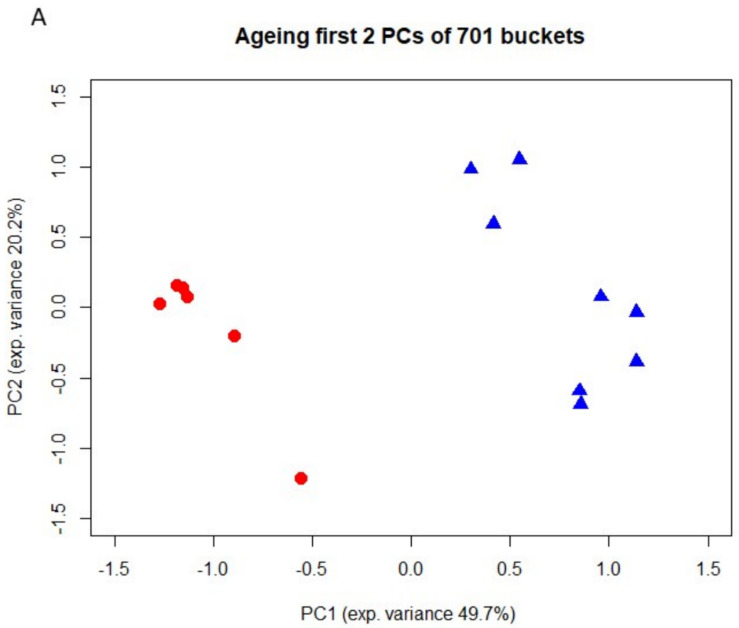

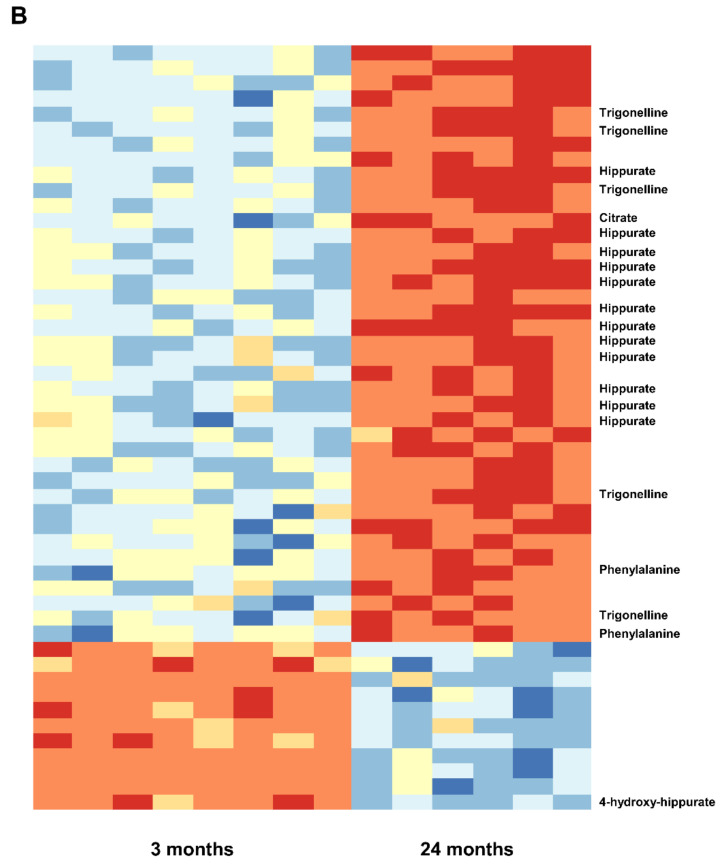
(**A**). Fingerprinting analysis from urine of 3- and 24-month-old rats by 1D 1H NMR spectroscopy. Principal component analysis (PCA). Blue triangels and red dots denote young and old animals, respectively. (**B**). Heatmap of the 50 most regulated features. Metabolites up- and down-regulated relative to their respective means are shown in blue and red, respectively. Samples 1–8 originate from young animals (3 months), while samples 9–14 correspond to old animals (24 months). Metabolites that could be unambiguously assigned to a given feature are marked.

To identify metabolites that might represent markers of ageing or kidney dysfunction, NMR spectroscopy was performed on urine samples from eight young and six old rats. First, 38 selected metabolites were quantified in either 1D or 2D NMR spectra. For twenty-one metabolites quantitative values could be determined in at least 10 out of a total of 14 spectra. To account for differences in fluid intake their levels were normalized to that of creatinine. For the identification of regulated metabolites a two-sided Welch test was performed, followed by controlling the false discovery rate to account for multiple testing. Results showed nine significantly regulated urine metabolites. Concentrations of hippurate, trigonelline, trimethylamine-N-oxide (TMAO), glutamine, 2-oxoglutarate (α-ketoglutarate), valine and citrate were significantly lower in urine of old rats, whereas acetoacetate and 1-methyl-histidine levels were significantly higher in old rats (Figure 2). Taurine was also increased in older rats, albeit not significantly (data not shown). Next, a non-targeted fingerprinting approach using all signals in the 1D spectra (but excluding urea- and water-specific signals) was performed as described in the Material and Methods subsection NMR spectroscopy. The first two components of a principal component analysis (PCA) clearly discriminated young from old rats (Figure 3A), indicating strong metabolic differences between the two age classes. For the identification of significantly regulated features a two-sided Welch t-test was performed. To account for multiple testing the false discovery rate was controlled at the 5% level according to the method of Benjamini and Hochberg leading to 320 significantly regulated features. The levels of the 50 most regulated features discriminating young from old animals are visualized in a heatmap representation (Figure 3B).

### 2.4. Renal Structure Alterations 

To identify age-related renal lesions that might serve as markers of kidney function, computer-aided histomorphometric analyses were conducted on kidney sections of 3- and 24-month-old rats.

**Figure 4:**
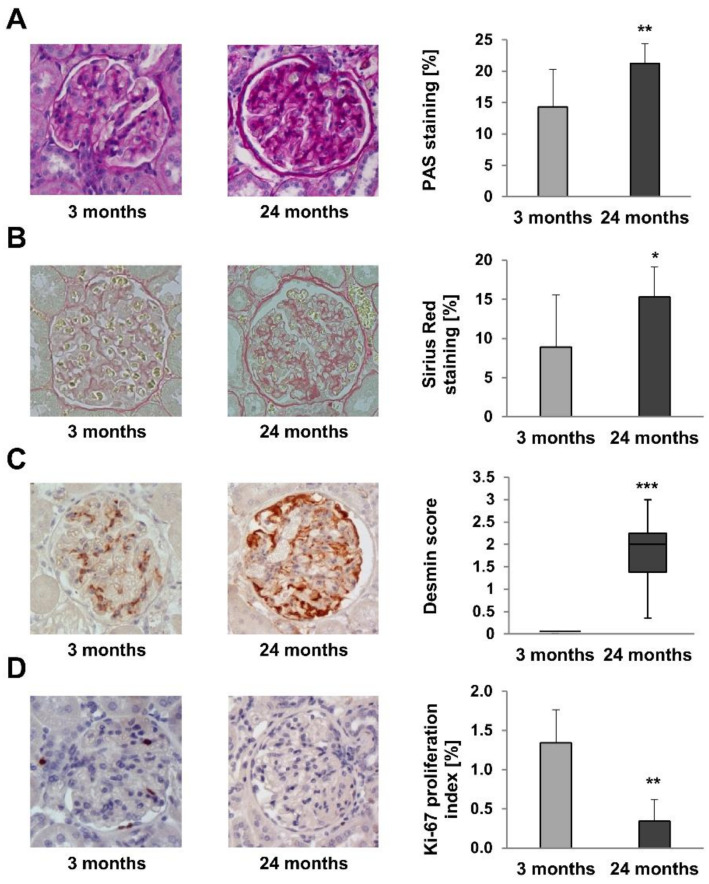
Histomorphometric analyses on kidney sections of 3- and 24-month-old rats. (**A**) PAS-positive areas were significantly increased in 24-month-old rats (*p* = 0.007). (**B**) Sirius Red staining of collagen, was significantly increased in 24-month-old rats (*p* = 0.033). (**C**) Glomerular desmin staining was significantly increased in 24-month-old rats (*p* = 0.001). (**D**) The proportion of Ki-67-positive proliferating glomerular cells was significantly reduced in 24-month-old rats (*p* = 0.002). For all Figures *p*-values ≤ 0.05, ≤0.01, and ≤0.001 are indicated by one, two or three asterisks, respectively. Magnification x400.

Glomerular deposition of PAS-positive extracellular matrix is a marker of renal function [19]. PAS-positive areas were significantly increased in 24-month-old rats (Figure 4A; *p* = 0.007). Similarly, Sirius Red staining of collagen, a marker of renal fibrosis [20], was increased in 24-month-old rats, too (Figure 4B; *p* = 0.033). Glomerular desmin staining, a marker of podocyte damage [21,22], was significantly increased in 24-month-old rats (Figure 4C; *p* = 0.001), while α-SMA staining, an indicator of mesangial cell proliferation and a proposed marker of age-related glomerular injury [23,24], was not significantly increased in older rats (data not shown; *p* = 0.169). In contrast, the proportion of Ki-67-positive proliferating glomerular cells was significantly reduced in 24-month-old rats (Figure 4D; *p* = 0.002). 

Electron microscopy revealed in comparison to young rats (Supplementary Figure S1) several renal lesions in 24-month-old rats, including focal or segmental effacement of foot processes, thickening of the glomerular basement membrane with focal subepithelial deposits, and low to moderate mesangial expansion in individual glomeruli, as well as tubular degeneration or atrophy and signs of interstitial fibrosis.

### 2.5. Renal Fibrosis Markers

**Figure 5:**
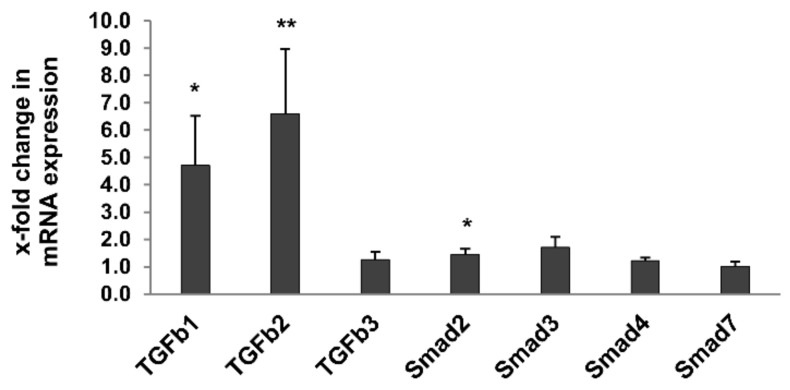
Expression of TGF-β pathway-specific genes in kidneys of 3- and 24-month-old rats by RT-qPCR. Expression of TGF-β1, TGF-β2 and Smad2 was significantly higher in 24-month-old rats compared to 3-month-old rats, while expression of TGF-β3, Smad3, Smad4 and Smad7 was not significantly affected. Data was normalized with respect to the corresponding values determined in 3-month-old rats. For all figures *p*-values ≤ 0.05, and ≤0.01 are indicated by one or two, respectively.

To further characterize markers of renal fibrosis, expression of TGF-β and Smad family members, known to be associated with progressive sclerosing injury in glomeruli and the tubulointerstitial compartment [25], were measured in the whole kidney using quantitative reverse transcription PCR (RT-qPCR). Expression of TGF-β1, TGF-β2 and Smad2 was significantly higher in 24-month-old rats compared to 3-month-old rats, while expression of TGF-β3, Smad3, Smad4 and Smad7 was not significantly affected (Figure 5).

### 2.6. Inflammaging Markers

Chronic inflammation and impaired immunity are hallmarks of ageing [9,15,16]. The characterization of age-related immune and inflammation markers is of particular importance in solid-organ transplantation, in the context of recipient immunosuppression [8]. Therefore, we evaluated the profiles of several immune and inflammation markers in 3- and 24-month-old rats.

**Figure 6:**
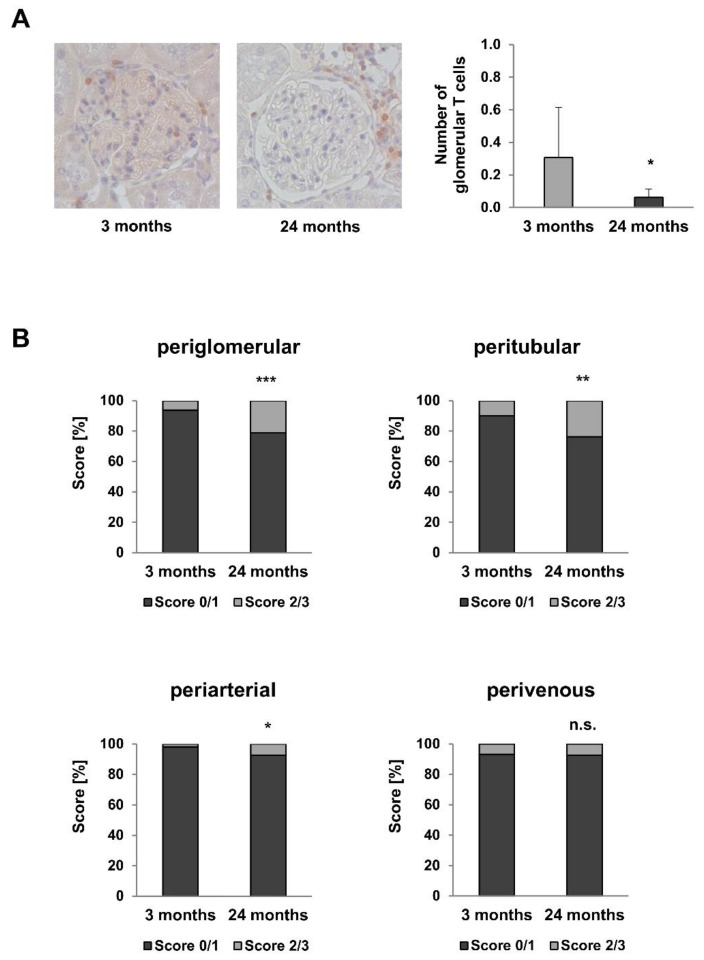
Glomerular and interstitial infiltration of T cells in 3- and 24-month-old rats. (**A**) Immunohistochemistry with CD3 antibody (magnification ×200). Intraglomerular infiltration of CD3-positive T cells was significantly reduced in 24-month-old rats (**B**). In contrast periglomerular, peritubular, and periarterial infiltration of T cells was significantly increased in 24-month-old rats. Perivenous T cell infiltration was also increased in old rats, albeit not significantly. For all Figures *p*-values ≤ 0.05, ≤0.01, and ≤0.001 are indicated by one, two or three asterisks, respectively.

Macrophages and T lymphocytes are important mediators of pro-inflammatory responses. Their respective numbers in 3- and 24-month-old rat kidneys were evaluated by immunohistochemistry. While the number of glomerular CD68-positive macrophages was comparable in young and old rats (1.52 vs. 1.49 per glomerulus, *p* = 0.599), intraglomerular infiltration of CD3-positive T cells was significantly reduced in 24-month-old rats (Figure 6A, *p* = 0.011). In contrast, interstitial infiltration of CD3-positive T cells (Figure 6B) was observed in older rats. More specifically, summarized scores 0/1 representing no or only a mild infiltration of CD3-positive T cells were significantly lower in 24-month-old rats. Here, the category “moderate or strong” infiltration (score 2/3) was more often assigned in periglomerular (*p* = 9.8 × 10^−5^), peritubular (*p* = 0.0012), and periarterial (*p* = 0.017) areas. Perivenous T cell infiltration exhibited no changes between both age groups.

**Figure 7:**
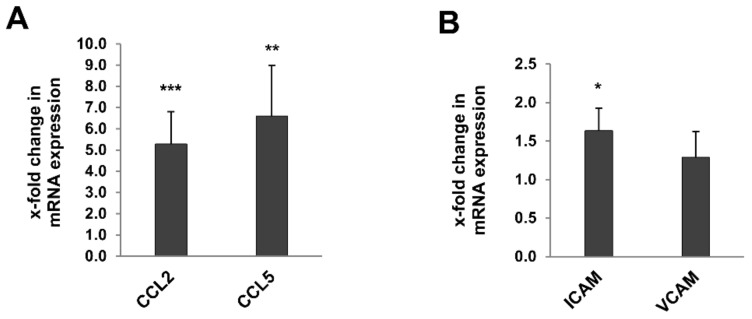
Expression of chemokines and adhesion marker genes in kidneys of 3- and 24-month-old rats by RT-qPCR. The expression of the chemokines CCL2 (MCP-1) and CCL5 (RANTES)(**A**) and the adhesion molecule ICAM1 (**B**) in the whole kidney of 24-month-old rats was significant increased, while expression of VCAM1 was comparable in 3- and 24-month-old rats (**A**,**B**). Data was normalized with respect to the corresponding values determined in 3-month-old rats. *p*-values ≤ 0.05, ≤ 0.01, and ≤0.001 are indicated by one, two or three asterisks, respectively.

Interstitial T cell infiltration correlated with a significant increase in the expression of the chemokines CCL2 (MCP-1) and CCL5 (RANTES; Figure 7A) and the adhesion molecule ICAM1 (Figure 7B) in the whole kidney of 24-month-old rats, while expression of VCAM1 was comparable in 3- and 24-month-old rats (Figure 7B).

**Figure 8:**
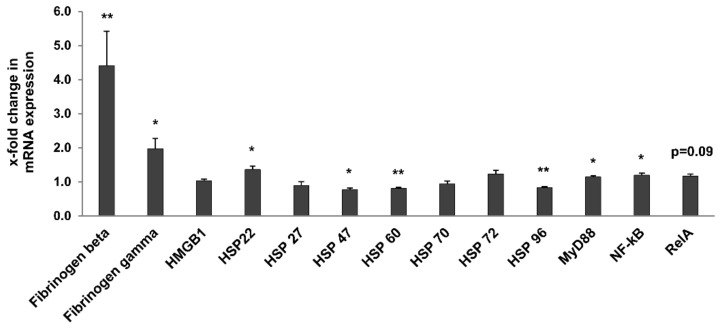
Expression of endogenous TLR ligands and downstream signaling molecules. Data was normalized with respect to the corresponding values determined in 3-month-old rats. *p*-values ≤ 0.05 and ≤0.01 are indicated by one or two asterisks, respectively.

The Toll-like receptor (TLR) and NF-κB signalling pathways are known for their role in the response to stress and inflammation and for their contribution to inflammaging [8,16,26]. We also showed that upregulation of some TLR members, notably TLR4, and of their endogenous ligands, such as fibrinogen, are associated to kidney damage [11,12]. Thus, we analysed the expression of TLR1–12 and some of their known endogenous ligands (Fibrinogen beta and gamma, HMGB1, HSPs) [26], as well as downstream signalling molecules (MyD88, NF-κB1, RelA) in whole kidneys of 3- and 24-month-old rats (Table 2, Figure 8). Most TLRs (apart from TLR2, TLR3 and TLR9; Table 2) were significantly upregulated in the kidney of old rats. TLR12 showed the highest upregulation (5.1-fold higher in 24- vs. 3-month-old rats; *p* = 0.007). Among the investigated endogenous ligands and signaling molecules, fibrinogens beta and gamma, HSP 22, NF-κB1, and myD88 were significantly upregulated (Figure 8), with fibrinogen beta showing the strongest increase (4.4-fold; *p* = 0.001) and MyD88 only a relatively slight increase in 24-month-old rats. HSP 47, HSP 60 and HSP 96 were significantly downregulated. Interestingly, TLR2, TLR3, TLR4, TLR6, and TLR7 were significantly upregulated in the liver of old rats, while only TLR8 was slightly but significantly upregulated in spleen (Table 2). In contrast, expression of TLRs was comparable in the lung of 3- and 24-month-old rats (Table 2).

### 2.7. Other Age-Related Renal Metabolic Markers

**Figure 9:**
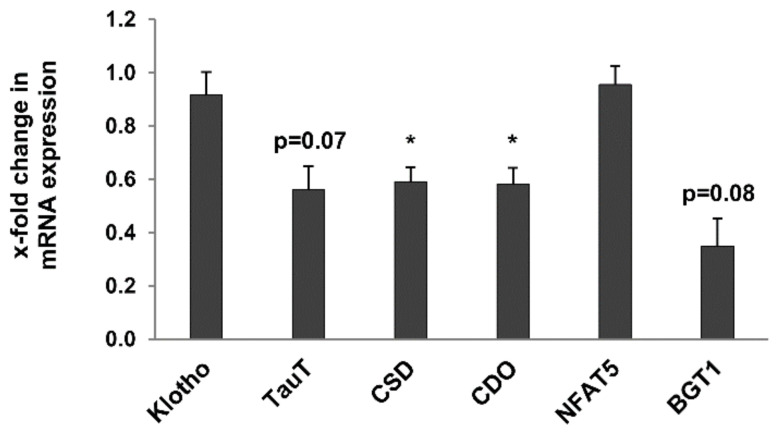
Expression of age-related renal metabolic markers in kidneys of 3- and 24-month-old rats by RT-qPCR expression of the anti-ageing kidney-secreted factor Klotho and of NFAT5, a transcription factor involved in the response to hypertonic and hypoxic stress in the kidney was not significantly altered in old rats. Expression of cysteine sulfinate decarboxylase (CSD) and cysteine dioxygenase (CDO), two enzymes involved in taurine biosynthesis, was significantly downregulated in 24-month-old rats. Similarly, expression of the taurine and betaine transporters, TauT and BGT1 respectively, was also reduced in old rats, albeit not significantly. Data was normalized with respect to the corresponding values determined in 3-month-old rats. *p*-values ≤ 0.05 are indicated by one asterisks.

Finally, the expression of genes known for their regulation of kidney metabolism and potential markers of kidney ageing was evaluated in whole kidneys of young and old rats. Expression of the anti-ageing kidney-secreted factor Klotho [27] and of NFAT5, a transcription factor involved in the response to hypertonic and hypoxic stress in the kidney [28] was not significantly altered in old rats (Figure 9). Expression of cysteine sulfinate decarboxylase (CSD) and cysteine dioxygenase (CDO), two enzymes involved in taurine biosynthesis, was significantly downregulated in 24-month-old rats (Figure 9). Similarly, expression of the taurine and betaine transporters, TauT and BGT1 respectively, was also reduced in old rats, albeit not significantly (Figure 9).

## 3. Discussion

This study aimed to characterize age-related markers of kidney function in a healthy rat model. These markers may be helpful to develop future biomarkers for a better evaluation of donor organ quality prior to kidney transplantation.

In line with previous studies, we demonstrated proteinuria (notably albuminuria) in old rats, together with unchanged blood pressure and serum creatinine levels [29]. The detection of albuminuria is in agreement with the apparent podocyte damage evidenced by the increased desmin immunostaining and the structural alterations of glomeruli identified by electron microscopy. Serum levels of glucose, BUN, and uric acid were lower in 24-month-old healthy rats, in agreement with previous observations in elderly humans. In this context, it is important to note that the increased levels of blood glucose often observed in elderly probands may be attributed to age-related disorders rather than age per se [30,31]. Of note, the use of male rats did not allow to evaluate the protein/creatinine ratio in urine [17], due to the presence of contaminating MUPs in young male animals [18]. The strong decrease in the glomerular proliferation index (Ki-67 staining) in old rats indicates age-related senescence [32].

Interstitial fibrosis was evidenced in old rats by Sirius Red staining and electron microscopy, and was supported by the upregulation of TGF-β1 and TGF-β2, and of Smad2 to some extent, as measured by RT-qPCR. Previous studies demonstrated interstitial immunostaining of TGF-β1 in ageing healthy Wistar rats [29]. The gene expression analysis performed on whole kidneys in our study does not allow to identify the kidney cell types expressing TGF-β family members. It would be interesting to expand this analysis, as glomerular and tubular expression of TGF-β1 and TGF-β2 has been shown to have antagonistic pro- or anti-fibrotic effects [25].

Several markers of inflammaging were identified in 24-month-old rats. Upstream and downstream signalling molecules of the TLR pathway were upregulated, including potential endogenous ligands (fibrinogen beta, fibrinogen gamma, HSP 22), most TLRs (TLR1, TLR4–8, TLR10–12), NF-κB1 and the adaptor molecule MyD88 and the downstream target genes CCL2 and CCL5. These observations are in line with previous studies of ageing in F344 rat kidneys [14]. These changes in gene expression correlated with the increased count of infiltrating interstitial T cells, which are key mediators of inflammation. Interestingly, several of these signaling molecules, notably TLR4, fibrinogen and MyD88 have been associated with impaired kidney function [5,11,13], suggesting that they might represent relevant markers of age-related kidney quality and function. It is worth noting that age-related changes in TLR gene expression were observed in kidney and liver, but not in the lung, suggesting that these inflammaging markers might not be applicable to all transplantable organs. 

The impact of inflammaging on kidney function is particularly critical in the context of recipient immunosuppression. Immunosuppressive drugs such as the calcineurin inhibitor cyclosporine promote, while mycophenolic acid-based immunosuppressors inhibit oxidative stress and inflammation [8]. Several randomized studies comparing the impact of immunosuppressive drugs have been performed [33,34,35,36]. Unfortunately, most randomized studies do not include very old patients (≥65 years and older) and more studies are needed to investigate the impact of adjusting immunosuppressive therapy and reducing nephrotoxic treatments on the long-term outcome of elderly recipients from old donor organs. In addition, the predictive value of inflammaging markers in donor organs pre-transplantation on post-transplantation outcome remains to be evaluated.

NMR spectroscopy applied on urine of young and old rats identified several metabolites whose levels relative to creatinine were altered with age. Similar age-associated changes in urinary concentration of 2-oxoglutarate, citrate, glutamine, hippurate TMAO, trigonelline, valine and taurine have been previously reported in rat and human [37,38,39,40]. Although not significant, it is interesting, that the increased urinary concentration of taurine in the urine of ageing rats correlates with a decreased expression of its transporter TauT in the kidney. This suggests that the increased urinary concentration of taurine might be the consequence of reduced renal tubular reabsorption rather than increased secretion. Also, increased urinary levels of 1-methyl-histidine and acetoacetate were observed for older rats. In elderly humans an increase in urinary ketone bodies has been linked to acute hyperglycemic crisis [41]. For the 1-methyl-histidine to creatinine ratio an increase with age has been previously reported, which might be explained by decreased creatinine levels at older age [42]. Data revealed in addition a decreased trigonelline to creatinine ratio for older rats. In contrast, Slupsky et al. had found for ageing humans an increase in urinary trigonelline [43], which is not surprising as trigonelline originates from dietary sources and, therefore, the observed differences are most probably dietary related. Given the non-invasive nature of the procedure and its potential amenability to routine application, NMR spectroscopy on urine samples of donors might be useful in case of living donation. Further investigations are needed to characterize and identify robust age-related markers of kidney function.

This study has several limitations. The size of the young and old rat population was small and the focus on male rats might have introduced a sex-specific bias (e.g., due to the presence of rodent-specific MUPs). In addition, because gene expression analyses were conducted on whole kidney tissue, they did not allow to identify cell-specific markers. Also, many components of the investigated inflammaging pathway are regulated through activation (e.g., phosphorylation) rather than through gene expression, thus age-related markers might not be best identified by RT-qPCR. On the other hand, the goal of our gene expression analyses was to identify markers with a strong differential expression between young and old rats, using a simple method (RT-qPCR) possibly amenable to routine investigation, for instance from kidney biopsies.

In conclusion, this study conducted in healthy male Sprague Dawley rats led to the identification of age-related changes of kidney function. Among those, markers of inflammaging and urine metabolites are the most promising and deserve further investigation in humans.

## 4. Materials and Methods

### 4.1. Animals and Tissues

Male Sprague Dawley rats were purchased from Janvier Labs (Saint Berthevin, France) and Charles River (Sulzfeld, Germany). Rats were fed a standard rodent diet (Ssniff R/MH, Soest, Germany) and had unlimited access to food and water. All animals were housed with a 12-h light/dark cycle in standard polycarbonate cages at 23 °C with a relative humidity of ∼50%. Overnight urine specimens were collected in metabolic cages at 3 and 24 months of age, centrifuged and stored at −20 °C until analysis. Animals were anesthetized and sacrificed at the age of 3 and 24 months (n = 8 per group), and blood and organs were collected. Blood was collected by cardiac puncture, centrifuged, and sera were stored at −20 °C. Both kidneys were perfused with 0.9% saline until the surface appeared pale. Portions of renal, liver, spleen and lung tissues were snap frozen in liquid nitrogen for further RNA isolation. A small portion of renal tissue was fixed in Karnovsky’s fixative (2% paraformaldehyde, 2.5% (*v/v*) glutaraldehyde, 100 mM cacodylate buffer [pH 7.4]) for electron microscopy analyses. Half of the remaining renal tissue was fixed in 10% neutral-buffered formalin for histology analyses and the other half was fixed in methacarn (60% vol/vol absolute methanol, 30% chloroform, 10% acetic acid) for immunohistochemistry analyses. 

### 4.2. Blood Pressure and Heart Rate

Blood pressure and heart rate were measured using the Coda-2 VPR noninvasive tail-cuff system (Kent Scientific, Torrington, CT, USA), in a quiet and warm environment. Blood pressure of each rat is the mean of 30 measurement cycles, subsequent to five acclimation cycles. 

### 4.3. Serum Analyses

Determination of serum levels of creatinine, blood urea nitrogen (BUN), uric acid, aspartate aminotransferase (ASAT), alanine aminotransferase (ALAT), total serum proteins, albumin, glucose, C-reactive protein (CRP), cholesterol, and triglycerides were kindly performed by the Institute of Clinical Chemistry and Laboratory Medicine of the University Hospital Regensburg using routine procedures. 

### 4.4. Analyses in Urine

BUN concentration in urine was kindly provided by the Institute of Clinical Chemistry and Laboratory Medicine of the University Hospital Regensburg applying routine procedures. 

Total protein concentration in urine was determined using an in-house Bicinchoninic Acid (BCA) assay. Ten μL of 1:10 urine were mixed with 200 μL of 0.08% Cu(II)SO4 (*w/v*) in bicinchoninic acid (Sigma-Aldrich, Taufkirchen, Germany) in a transparent flat-bottom 96-well plate (Nunclon, ThermoFisher Scientific, Waltham, MA, USA), and incubated for 1 h at 37 °C. Absorbance was read at 652 nm on the Infinite 200 PRO plate reader (Tecan, Männedorf, Switzerland).

Albumin concentration in urine was determined using the Mouse Albumin ELISA Quantification Set (Bethyl Laboratories, Montgomery, AL, USA), according to the manufacturer’s recommendations, from 1:50 and 1:200 diluted urine of 3- and 24-month-old rats, respectively. The Affinity-purified Mouse Albumin Coating Antibody was used 1:100 and the HRP-Conjugated Mouse Albumin Detection Antibody was used 1:40,000. 

Creatinine concentration in urine was measured in 10 μL 1:100 diluted urine using the Creatine PAP assay (Labor+Technik, LT-SYS, Eberhard Lehmann GmbH, Berlin, Germany), according to the manufacturer’s instructions. Urea concentration in urine was measured using the kit LT-UR010 (Labor+Technik, LT-SYS).

Proteinuria components were analysed by sodium dodecylsulfate polyacrylamide gel electrophoresis (SDS-PAGE). To this end, one μg of total protein was separated on an 8–18% gradient SDS-PAGE and visualized by silver staining, following standard protocols.

### 4.5. NMR-Spectroscopy

For NMR measurements 100 μL of urine were adjusted to a pH of 7.0 by either adding small amounts of 1N NaOH or 37% HCl followed by the addition of deionized water to give a total sample volume of 400 μL, which was mixed with 200 μL phosphate buffer, pH 7.4, and 50 μL D2O containing 0.75% (w) 3-trimethylsilyl-2,2,3,3-tetradeuteropropionate (TSP; Sigma-Aldrich, Taufkirchen, Germany) as internal standard. All NMR experiments were carried out on a 600 MHz Bruker Avance III spectrometer (Bruker BioSpin GmbH, Rheinstetten, Germany) using a triple-resonance (1H, 13C, 31P, 2H lock) cryo-probe with z-gradients. For each sample, a 1D 1H NMR spectrum and a 2D 1H-13C HSQC spectrum were obtained following established protocols [44].

Employing AMIX 3.9.13 (Bruker BioSpin, Ettlingen, Germany) assignment of metabolites was facilitated by comparison with reference spectra of pure compounds. A targeted analysis was performed for the quantification of a total of 38 preselected metabolites from both 1D and 2D NMR spectra. For the analysis of 2D spectra AMIX 3.9.13 (Bruker BioSpin) together with individual calibration factors for each metabolite signal was employed [44], while 1D spectra were quantified with Chenomx 8.6 (Chenomx Inc., Edmonton, AB, Canada). 

For non-targeted fingerprinting analysis of NMR data, bucket tables were generated from 1D 1H NMR spectra as described previously [45]. Briefly, the signal containing spectral region from 9.5−0.5 ppm was divided in evenly spaced bins of width 0.01 ppm. Excluding the broad urea signal and the region of the water artifact, 701 features were obtained per spectrum. Resulting tables were imported into the statistical-analysis software R version 4.0.3 (Development Core Team, R. R: A Language and Environment for Statistical Computing. (4.0.3). 2020. Vienna, Austria, R Foundation for Statistical Computing). Subsequent to normalization with respect to creatinine, for both targeted and non-targeted data, significant differences between groups were assessed based on the Welch t-test. To account for multiple testing the false discovery rate was controlled at the 5% level according to the method proposed by Benjamini and Hochberg [46]. For visual inspection of group differences principal component analyses (PCA) and heatmaps were generated within R. 

### 4.6. Tissue Preparation and Histologic Staining 

Formalin-fixed tissues were embedded in paraffin, sectioned (1 and 3 µm) and stained with Sirius red (1 µm sections) or with periodic acid-Schiff (PAS; 3 µm sections) reagents following standard protocols. Pictures of 20 randomly selected glomeruli were taken using AxioCam ICc1 with the AxioVision software on a Axiostar Plus microscope (Carl Zeiss, Jena, Germany). PAS and Sirius Red stained areas were enclosed with a color threshold and referred to total glomerular area using the HistoQuest software (TissueGnostics GmbH, Vienna, Austria). 

### 4.7. Immunohistochemistry and Morphometric Analyses

Methacarn-fixed kidneys were embedded in paraffin, sectioned (3 µm), and stained using the avidin-biotin complex (ABC) method, as previously described [11,20]. Antigen retrieval was done by steam-heating using a decloaking chamber pro (Biocare Medical, Concord, CA, USA). Sections were incubated with the primary antibodies overnight at 4 °C and with the biotin-conjugated secondary antibodies for one hour at room temperature. The following primary antibodies and dilutions were used: mouse anti-rat Ki-67 (M7248, Dako; 1:25), mouse anti-rat α-smooth muscle actin (α-SMA; A2547, Sigma; 1:200), mouse anti-rat CD68 (MCA3476A9, Bio-Rad; 1:500), rabbit anti-rat CD3 (ab5690, abcam; 1:100) and mouse anti-rat Desmin (M0760, Dako; 1:100). Anti-mouse (1:500) and anti-rabbit (1:300) secondary antibodies were from Jackson ImmunoLab (715-065-151 and 711-065-152, respectively). For each specific staining, a mouse or rabbit isotype (IgG) condition (in place of the primary antibody) was used as a negative control. Immunostained sections were counterstained with hematoxylin.

Morphometry of immunostained sections was performed by an observer blinded to their origin. For each slide, pictures of 20 randomly selected glomeruli were taken using a Zeiss digital camera (Axiocam ICc1 and AxioVision Software) on a Axiostar Plus microscope (Carl Zeiss, Jena, Germany). CD68-, CD3- and Ki-67-positive cells were enumerated using the HistoQuest software (TissueGnostics GmbH, Vienna, Austria) and respective arithmetic means (±SD) were calculated. Ki-67-positive cell counts were expressed as a percentage of the total number of glomerular cells. The scoring system for CD3-positive cells (0 to 3) was extended to evaluate the interstitial kidney compartment, including the periglomerular, -arterial, -venous, and -tubular areas. CD3 staining was scored as follows: no staining (score 0), isolated stained cells (score 1), multiple clusters of stained cells (score 2), and strong or continuous staining around structures (score 3). α-SMA staining in mesangium was scored (0 to 4) according to Johnson et al. [21], as follows: no staining in glomerulus (score 0), segmental staining in the mesangial area (score 1), staining outlines the mesangial stalk (score 2), staining of the mesangial stalk with focal areas of nodularity (score 3), and staining of the entire glomerulus with diffuse areas of nodularity (score 4). Desmin staining of the outer edge of the glomerular tuft was scored (0 to 4) according to the percentage of outer edge with positive staining [22], as follows: 0 to 5% stained (score 0), 5% to 25% stained (score 1), 25% to 50% stained (score 2), 50% to 75% stained (score 3), and ≥75% stained (score 4). 

### 4.8. Electron Microscopy

Karnovsky-fixed kidney tissue samples were processed, sectioned (80 nm), and examined by electron microscopy according to standard protocols [23], using a LEO912AB electron microscope (Carl Zeiss, Jena, Germany). 

### 4.9. RNA Isolation and Quantitative RT-PCR

Extraction of total RNA from rat tissues (kidney, liver, spleen, and lung) was performed using the RNeasy Midi Kit (Qiagen, Hilden, Germany) followed by DNase I (79254, Qiagen) treatment. One µg total RNA was used for cDNA synthesis with random primers and 200 U murine Molony leukemia virus (M-MLV) reverse transcriptase (Promega, Madison, WI, USA), and 13 ng RNA equivalent was used per quantitative PCR (qPCR) reaction. qPCR reactions (10 µL final) were performed in triplicate in 384-well plates using QuantiTect Sybr Green PCR Master Mix (Qiagen, Hilden, Germany) and the 7900HT Fast Real-Time PCR System (Applied Biosystems, Darmstadt, Germany). The thermal protocol comprised 15 min activation at 95 °C followed by 40 cycles of the following 3-step PCR conditions: 15 s denaturation at 95 °C, 30 s annealing at 58 °C and 30 s extension at 72 °C. PCR primers were designed using NCBI Primer-BLAST (Supplementary Table S1) and ordered from Eurofins (Ebersberg, Germany). Cyclophilin was used as a reference gene. No-template controls (ddH2O) were included and were negative in all cases.

Relative quantitation of gene expression in 3-month-old and 24-month-old rat kidneys was performed using the ΔΔCt method. qPCR data were normalized to the reference gene cyclophilin and expressed as fold change relative to the 3-month-old expression level (mean fold change of triplicate PCR = 1).

### 4.10. Statistical Analysis

Differences between groups (3-month-old vs. 24-month-old rats) were tested using either a two-sided parametric student’s *T*-test for metric data (albumin/protein level, metabolites, morphometric, cell count, and qPCR analyses) or a two-sided non-parametric Mann-Whitney U-test for ranked data (desmin staining, α-SMA staining). *p*-values < 0.05 were considered statistically significant. For group comparison after CD3 staining, scores of 0 and 1 were summarized as the category “no or mild staining” and the scores 2 and 3 as “moderate or strong staining”. Numbers of allocated scores in both categories were analyzed for statistical significance applying a chi-square test with a significance level of *p* < 0.05. 

## Figures and Tables

**Table 1:** Male Sprague Dawley Rats’ characteristics at 3 and 24 months of age.

Parameters	3 Months	24 Months	*p*-Value (MWU)
Vital parameters			
Systolic blood pressure (mmHg)	159 (±14)	154 (±24)	0.721
Diastolic blood pressure (mmHg)	111 (±15)	105 (±19)	0.674
Heart rate (beats/min)	490 (±38)	499 (±47)	0.721
Serum parameters			
Creatinine (mg/dL)	0.32 (±0.03)	0.28 (±0.03)	0.051
Albumin (g/L)	12.5± (1.4)	11.9 (±1.3)	0.598
Total protein (g/L)	58.6 (±4.3)	68.3 (±1.8)	0.793
Blood urea nitrogen (BUN) (mg/dL)	50 (±5.8)	21 (±4.4)	0.001
Uric acid (mg/dL)	3.4 (±0.5)	0.4 (±0.1)	0.002
C-reactive protein (CRP) (mg/dL)	<2.9	<2.9	-
ASAT (mg/dL)	143 (±44.5)	103 (±20.3)	0.065
ALAT (mg/dL)	62.5 (±7.6)	43.3 (±22.8)	0.052
Glucose (mg/dL)	151 (±37.4)	80 (±20.6)	0.001
Cholesterol (mg/dL)	92 (±12.7)	105 (±38.1)	0.753
Triglycerides (mg/dL)	139 (±52.9)	156 (±92.1)	0.958

**Table 2:** Expression of TLR1–12 in kidney, liver, spleen and lung of 24-month-old rats expressed as fold change relative to 3-month-old rats. *p*-values ≤ 0.05, ≤0.01, and ≤0.001 are indicated by one, two or three asterisks, respectively.

**TLR**	**Kidney**	**Liver**	**Spleen**	**Lung**
	FC at 24 Months Relative to 3 Months	FC at 24 Months Relative to 3 Months	FC at 24 Months Relative to 3 Months	FC at 24 Months Relative to 3 Months
**TLR1**	2.02 (±1.07) *p* = 0.0023 **	0.85 (±0.64) *p* = 0.8785	0.78 (±0.58) *p* = 0.3357	0.78 (±0.51) *p* = 0.4634
**TLR2**	1.74 (±1.17) *p* = 0.0728	1.93 (±0.80) *p* = 0.0019 **	1.01 (±0.57) *p* = 0.2319	1.15 (±0.41) *p* = 0.3969
**TLR3**	0.96 (±0.36) *p* = 0.9015	2.64 (±0.96) *p* = 0.0002 ***	1.10 (±0.69) *p* = 0.6126	1.07 (±0.48) *p* = 0.6943
**TLR4**	1.72 (±0.73) *p* = 0.0041 **	1.64 (±0.63) *p* = 0.0047 **	1.10 (±0.73) *p* = 0.0541	0.99 (±0.28) *p* = 0.6943
**TLR5**	3.78 (±6.05) *p* = 0.0379 *	2.19 (±3.46) *p* = 0.4005	1.23 (±1.56) *p* = 0.6126	1.14 (±1.31) *p* = 0.7789
**TLR6**	1.45 (±0.42) *p* = 0.0023 **	1.86 (±0.69) *p* = 0.0019 **	0.80 (±0.51) *p* = 0.8665	1.10 (±0.35) *p* = 0.6943
**TLR7**	1.95 (±1.23) *p* = 0.0379 *	2.40 (±1.02) *p* = 0.0002 ***	1.43 (±0.55) *p* = 0.0939	1.01 (±0.34) *p* = 0.8665
**TLR8**	2.72 (±2.14) *p* = 0.0041 **	1.34 (±0.56) *p* = 0.0830	1.48 (±0.49) *p* = 0.0140 *	1.07 (±0.44) *p* = 0.6126
**TLR9**	2.01 (±3.69) *p* = 0.1282	2.80 (±7.56) *p* = 0.1949	1.05 (±2.39) *p* = 0.8665	1.01 (±2.38) *p* = 0.8665
**TLR10**	3.89 (±3.40) *p* = 0.0006 ***	1.04 (±0.55) *p* = 0.8785	0.89 (±0.78) *p* = 0.3357	0.75 (±0.70) *p* = 0.5358
**TLR11**	2.73 (±1.80) *p* = 0.0012 **	1.00 (±0.58) *p* = 0.9591	0.77 (±0.56) *p* = 0.3969	0.57 (±0.50) *p* = 0.1520
**TLR12**	5.09 (±4.66) *p* = 0.0070 **	1.07 (±0.67) *p* = 0.8785	0.73 (±0.58) *p* = 0.4634	0.74 (±0.55) *p* = 0.3969

## Data Availability

The data underlying this article are available in the article and in its online supplementary materials.

## Supplementary Materials

The following supporting information can be downloaded at: https://www.mdpi.com/article/10.3390/metabo12020162/s1, Table S1. Forward and reverse primers used for RT-qPCR. Figure S1. Electron microscopy of kidneys of 3- and 24-month-old rats.
