# Supplementary material for: Assessment of Physiological Rat Kidney Ageing—Implications for the Evaluation of Allograft Quality Prior to Renal Transplantation
Source: Metabolites. 2022 Feb 8;12(2):162. doi: 10.3390/metabo12020162 (PMC8875225; doi:10.3390/metabo12020162)
Supplement: Supplementary file 1 [file metabolites-12-00162-s001.zip › metabolites-1508377-supplementary.pdf]

Supplementary data

Table S1. Forward and reverse primers used for RT-qPCR

Gen	forward-Primer	reverse-Primer
TGF-β1	5'-gcggactactacgccaaaga-3	5'-actgcttcccgaatgtctga-3
TGF-β2	5'-cgaggagtactacgccaagg-3	5'-aggtacccacagagcacctg-3
TGF-β3	5'-ggagttgctggaagagatgc-3	5'-gtaattcccttggggcagac-3
Smad2	5'-ccaggtctcttgatggtcgt-3	5'-gagctcatgatggctgtgaa-3
Smad3	5'-cattaccatccccaggtcac-3	5'-tgttgaaggcgaactcacag-3
Smad4	5'-tcagtctgtctgctgctgct-3	5'-cggtggaggtgaatctcaat-3
Smad7	5'-ccaactgcagactgtccaga-3	5'-caggctccagaagaagttgg-3
TLR1	5'-tcttaccctgaacaacgtggacac-3	5'-aagcatgtggaccatgcgtgttc-3
TLR2	5'-aagaggaagcccaagaaagc-3	5'-tgaagggtgggtcagagttc-3
TLR3	5'-cttgcggtactgctcattca-3	5'-ctgctcaccctgtgcatcta-3
TLR4	5'-cgctctggcatcatcttcat-3	5'-ctcctcaggtcaaagttgttgc-3
TLR5	5'-ggatcatggcgtatcagctt-3	5'-acgatgtggtgacaaccgta-3
TLR6	5'-ggctggcctgactcttacag-3	5'-cagccagtagcagcagagtg-3
TLR7	5'-atgccaccgaatctaacgac-3	5'-gttgtggctgaggtccaaat-3
TLR8	5'-tggacttggggagaaatgag-3	5'-aagccagcaggtaggtgaga-3
TLR9	5'-agcactcccgtctcaaagaa-3	5'-tgacgaacatctctggcttg-3
TLR10	5'-ctgtgaggagattggcaaca-3	5'-gcaggctgccttcttcatag-3
TLR11	5'-ggttcctactctgccagcaa-3	5'-ttggcaagtagatgctgtgg-3
TLR12	5'-gcccatgtatctgaccagcttagag-3	5'-actgaagtttggggagctgccaga-3
CCL2	5'-atgcagttaatgccccactc-3	5'-ttccttattggggtcagcac-3
CCL5	5'-atatggctcggacaccactc-3	5'-tgacaaagacgactgcaagg-3
ICAM	5'-tgtgtattcgttcccagagc-3	5'-actactgagagctgtgtccg-3
VCAM	5'-ggagtgaatctggttgggag-3	5'-tgtcagaacaacggaatccc-3
MyD88	5'-atgaactgaaggaccgcatc-3	5'-gatgcctcccagttcctttg-3
NF-κB1	5'-ggcagacgacgatcctttc-3	5'-ggtatgggccatctgttgac-3
RelA	5'-tcagtgagcccatggagtcc-3	5'-catgatgctcttgaaggtctcg-3
Fibrinogen beta	5'-cctacgacagggacaacgat-3	5'-tgtaaaggccaccccagtag-3
Fibrinogen gamma	5'-gtgcgaatccatgacacaac-3	5'-tttccagatccgtcgatttc-3
HSP22	5'-caagccggaagaactgatgg-3	5'-ctggatccacttctgcagga-3
HSP27	5'-agctcacagtgaagaccaagg-3	5'-aagcaccgagagatgtagcc-3
HSP47	5'-gggcactgctcgttaatgc-3	5'-ggtgcatcatcgtaacaccc-3
HSP60	5'-gagaagatcagcaaaggggc-3	5'-tcaggggttgtcacaggttt-3
HSP70	5'-agggtctcaagggcaagatc-3	5'-ttctcagccagcgtgttaga-3

HSP72	5'-cgctccaggtgtgatctagg-3	5'-ttgcagaccgaacgaaggag-3
HSP96	5'-gcaacccagatgtcttggtg-3	5'-atgacagtgccctcttctcc-3
HMGB1	5'-tgattaatgaatgagttcgggc-3	5'-tgctcaggaaacttgactgttt-3
Klotho	5'-gatgttcgtgacagccaatg-3	5'-cgcaaagtagccacaaaggt-3
TauT	5'-attgtcatcctcctctgccg-3	5'-tctggagtgaaagggcgtag-3
CDO	5'-tgcctttgaccaacgaacag-3	5'-ggcttggcaggtcttagttg-3
CSD	5'-cgcatcattacggagagcc-3	5'-agggcacggagtttcttcag-3
NFAT5	5'-ttaccacggacaacaaaggc-3	5'-cttcagctccttcccctcac-3
BGT1	5'-tctctgctacaagaacgggg-3	5'-ctggctgctatactgaccca-3

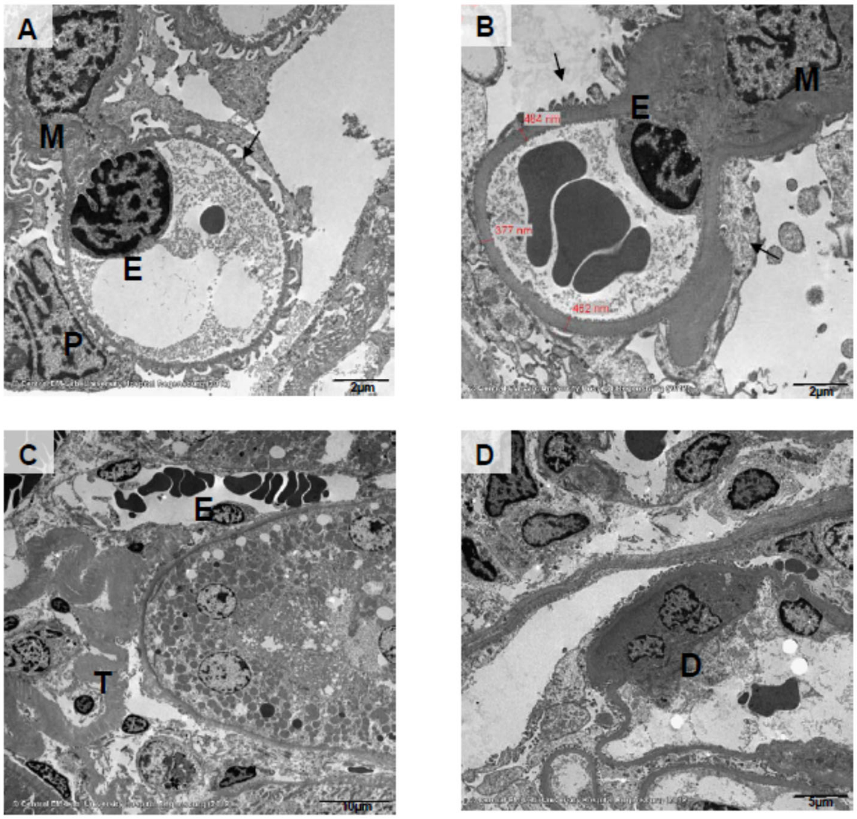

Figure S1. Electron microscopy of kidneys of 3- and 24-month-old rats. (A) Glomerular capillary of a 3-month-old rat covered by regular podocyte foot processes, **(B)** Glomerular capillary of a 24-month-old rat, showing a thickening of the glomerular basement membrane, a focal subepithelial deposit, and segmental effacement of podocyte foot processes; **(C)** degenerated tubule in the interstitium of a 24-month-old rat. The collapsed tubule wall shows hardly any epithelial cells; **(D)** focal membranous glomerular deposits and perivascular fibrocytes in the interstitium of a 24-month-old rat, indicative of interstitial fibrosis. Legend: E, endothelial cell; D, deposit; M, mesangial cell; T, tubule; arrow, points to podocyte foot processes. Size scale: bar A and B = 2μm, bar C = 10 μm, D = 5μm.
